# Principles of phosphoproteomics and applications in cancer research

**DOI:** 10.1042/BCJ20220220

**Published:** 2023-03-24

**Authors:** Luke Higgins, Henry Gerdes, Pedro R. Cutillas

**Affiliations:** 1 Cell Signaling and Proteomics Group, Centre for Genomics and Computational Biology, Barts Cancer Institute, Queen Mary University of London, London, U.K.; 2 Alan Turing Institute, The British Library, London, U.K.; 3 Digital Environment Research Institute, Queen Mary University of London, London, U.K.

**Keywords:** kinase biology, mass spectrometry, proteomics

## Abstract

Phosphorylation constitutes the most common and best-studied regulatory post-translational modification in biological systems and archetypal signalling pathways driven by protein and lipid kinases are disrupted in essentially all cancer types. Thus, the study of the phosphoproteome stands to provide unique biological information on signalling pathway activity and on kinase network circuitry that is not captured by genetic or transcriptomic technologies. Here, we discuss the methods and tools used in phosphoproteomics and highlight how this technique has been used, and can be used in the future, for cancer research. Challenges still exist in mass spectrometry phosphoproteomics and in the software required to provide biological information from these datasets. Nevertheless, improvements in mass spectrometers with enhanced scan rates, separation capabilities and sensitivity, in biochemical methods for sample preparation and in computational pipelines are enabling an increasingly deep analysis of the phosphoproteome, where previous bottlenecks in data acquisition, processing and interpretation are being relieved. These powerful hardware and algorithmic innovations are not only providing exciting new mechanistic insights into tumour biology, from where new drug targets may be derived, but are also leading to the discovery of phosphoproteins as mediators of drug sensitivity and resistance and as classifiers of disease subtypes. These studies are, therefore, uncovering phosphoproteins as a new generation of disruptive biomarkers to improve personalised anti-cancer therapies.

## Introduction

Cancer is caused by mutations in genes that, in normal cells, regulate fundamental cell biological processes such as bioenergetic metabolism, apoptosis and lineage identity [1]. However, genetic mutations, copy number alterations and transcriptional over/under-expression, while important, are not the only mechanisms by which the biological activities of oncogenes and tumour suppressor genes are dysregulated in cancer cells [2]. Virtually all druggable oncogenic processes are controlled by signalling networks regulated by post-translational protein modifications (PTMs), the most common and best studied of which is the addition of a phosphate group to serine, threonine or tyrosine residues, although other amino acids can also be phosphorylated [3]. Protein phosphorylation, which is catalysed by protein kinases and opposed by protein phosphatases, cooperates with other regulatory mechanisms (e.g. second messengers and protein–protein interactions (PPIs)) and enzymes (e.g. GTPases, phosphatases and lipid kinases), which together regulate intracellular signalling fluxes and virtually all cell biological functions. In normal cells, these kinase-driven signalling pathways are activated by extracellular signals provided by environmental cues (e.g. growth factors and hormones), direct cell–cell interactions and by mechanical and other stresses. In cancer, these pathways may be aberrantly activated as a result of genetic and epigenetic alterations. In addition, the microenvironment is often corrupted with the infiltration of immune and aberrant mesenchymal cells [4,5]. This tumour-associated stroma activates kinase signalling pathways in neighbouring cancer cells [6,7] and further influences their malignant phenotype which becomes imprinted through epigenetic mechanisms [8,9]. Thus heterotypic communication between cancer and stromal cells, which together make up a tumour, can lead to the constitutive activation of oncogenic signalling pathways in the absence of genetic alterations [6,7]. In immunologically ‘cold' tumours such as prostate cancer, dysregulated kinase signalling can occur due to genetic and/or epigenetic alterations that activate oncogenic drivers, thus representing tangible therapeutic targets with potential for precision medicine approaches [10].

The range and heterogeneity of molecular mechanisms that can lead to oncogenic pathway activation make the study of the phosphoproteome critical for a comprehensive understanding of cancer biology. Indeed, since each phosphorylation is, by definition, the product of kinase activity, the phosphoproteome provides readouts of all kinase-driven pathway activities in a given tumour. Thus, phosphoproteomic data capture oncogenic pathway activation irrespective of the mechanism by which such activation occurred — genetic, epigenetic or micro-environmental [11].

By providing a more direct measurement of cell processes, phosphoproteomic characterisation is enabling the identification of new pharmacological anti-cancer targets. In addition, quantification of phosphosites is being used to identify disease subgroups not captured by genetic assays [12–14], and to investigate the mechanisms of intrinsic [15–17] and acquired [18–20] resistance to targeted drugs, thus potentially leading to more effective personalised anti-cancer therapies [14,20–28].

The importance that the phosphoproteome has to illuminate oncogenic signalling has spurred intense research into technological development for its characterisation. To highlight the magnitude of the challenge, it has been estimated that the human phosphoproteome contains 13 000 phosphoproteins and 230 000 phosphorylation sites [29], most of which are present at substoichiometric amounts and are thus undetectable by standard mass spectrometry proteomics methods (i.e. without phosphoenrichment). Another issue is that most of these sites are not annotated with functional information or with the kinase(s) that catalyse their incorporation on proteins. The study of the phosphoproteome is also obscured by prolific kinase interactivity and rapid signalling dynamics. To untangle this, improvements in scan rates in modern mass spectrometers and refined sample preparation techniques have increased the depth, throughput and profiling capabilities of mass spectrometry proteomics/phosphoproteomics [30]. This, together with the development of computational concepts for the analysis and biological interpretation of phosphoproteomics data, is currently leading to a boom in the use of this technique for cancer kinome profiling.

Here, we review the current state of phosphoproteomics in cancer research with a focus on mass spectrometry-based approaches, and on introducing core concepts to new users, including methodologies and challenges that the field still faces. Finally, we review phosphoproteomic studies that illustrate how this technique is contributing, and can contribute in the future, to our understanding of cancer cell signalling and biology.

## Methods for phosphoproteomic interrogation of cancer

### Mass spectrometry interrogation of the proteome/phosphoproteome

Whilst immunoassays, such as reverse phase protein arrays (RPPA), allow the detection of 100s of analytes, it is easy to recognise the exciting potential of untargeted mass spectrometry-based methods which allow the quantification of 10 000s of phosphorylation sites in an untargeted manner. Indeed, it is using these technologies we now appreciate the complexity and heterogeneity of proteomic/phosphoproteomic profiles and signalling circuitries, with specific variations noted between patients and even cells [31–33].

**Figure 1.**
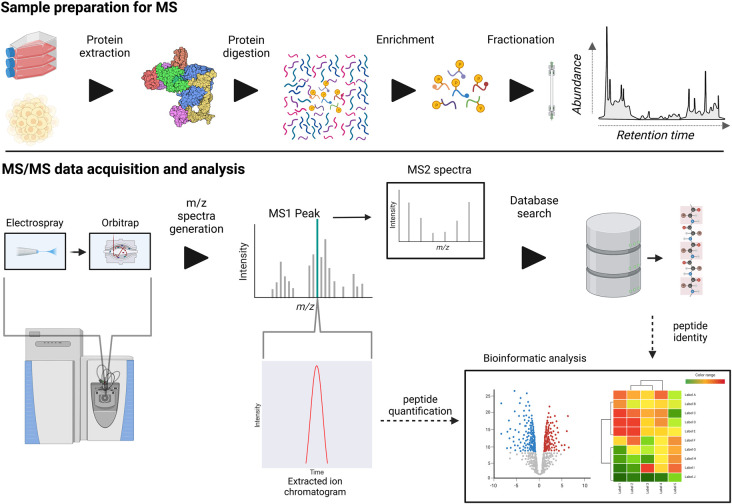
Liquid chromatography-tandem mass spectrometry (LC–MS/MS) workflow for data-dependent analysis (DDA) phosphoproteomics. Upper panel, phosphoproteomic sample preparation. Proteins are extracted from samples through cell lysis and digested using proteases, commonly trypsin. Peptide fraction is subsequently enriched for phosphopeptides, conventionally using TiO_2_, and separated using reversed-phase liquid chromatography before MS/MS analysis. Lower panel, MS/MS data acquisition and analysis. Following liquid chromatography, eluting peptides are ionised by electrospray and sampled by Orbitrap or TOF to generate MS1 spectra. Precursor ions are then selected and then fragmented. MS2 spectra are then generated from the second round of mass analysis. MS1 peaks can be isolated (extracted ion chromatogram) and used for quantification, whilst MS2 spectra enables peptide identification.

At the simplest level, mass spectrometry is the measurement of the mass-to-charge ratio (*m*/*z*) of ions in the gas phase. The acquired *m*/*z* spectra are used to infer the analyte constitution. For the study of the phosphoproteome/proteome ‘shotgun' or ‘bottom-up' proteomics is most conventionally used and currently provides the most comprehensive profiling approach. In brief, proteins/phosphoproteins are digested into peptides (some of which will be post-translationally modified), separated using chromatography and sampled into the mass spectrometer (Figure 1). Whilst mass spectrometry analysis of whole proteins is possible, a method named top-down proteomics, approaches for the analysis of undigested proteins are immature compared with bottom-up methods as these suffer from complications that arise from chromatography of native proteins, decreased sensitivity and less developed bioinformatics approaches [34].

Bottom-up phosphoproteomics requires the use of high resolution and high mass accuracy mass spectrometry provided by time of flight (TOF) or Orbitrap instruments. TOF mass analysers use the ion's deflection time as the informant of it mass. On the other hand, the Orbitrap mass analyser obtains ion m/z by tracking ion orbit frequency around the central electrode [35]. In proteomics/phosphoproteomics, these instruments are conventionally used with other ion optics that select and fragment peptide ions for tandem mass spectrometry (MS/MS or MS2) analysis. In this, a MS1 scan is used to obtain the mass of unfragmented peptides and for their quantification (in the case of conventional label-free analysis). Ions are then selected for subsequent fragmentation generating MS2 data used to infer the primary structure of peptides (Figure 1, lower panel). Peptide precursor ion *m*/*z* ratio in MS1 and MS2 fragment spectra allows for accurate identification of phosphopeptides.

Many fragmentation methods exist for MS2 spectra generation and the development of such tools still is an active area of research [36]. Collision-induced dissociation (CID), which applies gas-phase collision with neutral gas molecules to fragment gas-phase peptide ions, is the standard fragmentation method present in essentially all commercial tandem mass spectrometers. Therefore, CID has been widely adopted for phosphoproteomics research. Whilst in general efficient, ‘neutral loss' (i.e. loss of phosphate in the phosphorylated peptide fragment ions during CID) can obscure precise identification of the phosphorylated site in cases where more than one phosphorylatable amino acid residue is present in the peptide's sequence [37]. To address this, inspirited by electron-capture dissociation (ECD) which preserve the site of modification [38], electron transfer dissociation (ETD) was developed and incorporated into tandem mass spectrometers based on ion traps. These softer fragmentation methods are based on the transfer of electrons from radical anions to the polypeptide chain which as a consequence dissociate at the C–N bond [39,40]. ETD is also particularly useful for top- and middle-down proteomics used to characterise large proteins without or with just partial digestion [41,42] and the technique is particularly useful for the analysis of the histone PTMs [43]. ETD has been combined with higher-energy collision dissociation (HCD), a technique known as EThcD, by applying HCD following an electron transfer reaction [44], and this technique is particularly useful for the analysis of large proteins including membrane proteins [45].

Quantification from mass spectrometry data may be achieved using label-based or label-free approaches. Tandem mass tag (TMT) labelling uses isobaric labels consisting of heavy stable isotopes, and enables sample multiplexing which can expedite mass spectrometry analysis by quantifying reported ions in MS2 data [46]. Another labelling strategy consists of stable isotope labelling by/with amino acids in cell culture (SILAC) involving the culture of cells in media containing labelled amino acids [47]. A limitation of label-based approaches is that there is a limit to the number of samples and replicates that can be analysed in an experiment and that quantification is always relative to analytes present in other samples, thus limiting their utility for translational research and ultimately clinical implementation. Thus, label-free quantification has proven popular in phosphoproteomics, after showing that these approaches are effective for quantitative analysis of phosphopeptides [48–50], although a limitation has been the requirement of specialised data acquisition strategies and software.

### Sample preparation strategies for label-free phosphoproteomics

Phosphoproteomic experiments require phosphopeptide enrichment methods to reduce complexity and maximise deconvolution whilst retaining the biological information held within the sample [51]. Thus, much effort in the field has strived towards optimised sample processing procedures which do not influence the phosphoproteome before analysis or compromise profiling depth [52].

As mentioned, shotgun proteomics requires proteins extracted from samples to undergo digestion into peptides using peptidases such as trypsin, but others are available and can yield different peptide pools. Logically, the choice of digestive enzyme will influence the resulting peptide pool due to its cleavage site specificity [30]. However, phosphorylated residues can impact possible cleavage sites and thus impact digestion and detectability due to peptide length and ionisation efficiency [53]. Online tools which predict protein cleavage are able to assist in the making this decision, such as PeptideCutter [54].

Following digestion, phosphopeptide enrichment steps are used to increase the proportion of phosphopeptides identified in an experiment. The most popular phosphopeptide isolation methods take advantage of the negative ionic charge of the phosphate group. Immobilised metal affinity chromatography (IMAC) uses an affinity-based phenomenon, where the negative charge of phosphate groups is exposed to metal cations in the form of chelators. With potentially decreased specificity in comparison to other affinity methods, its applicability can be broad [55]. Drawbacks of this method include the risk of phosphopeptide leaching and binding of acidic amino acids such as glutamic and aspartic acid [55].

Metal oxide affinity chromatography (MOAC) applies the same affinity-based property as IMAC. Here, phosphopeptides are extracted by ionic binding between metal oxides and the negatively charged phosphate groups. Most commonly, titanium dioxide (TiO_2_) is the agent used for this approach, although, a range of metal oxides are available, Aryal and Ross demonstrated that between ZrO_2_ and TiO_2_, TiO_2_ displayed better selectivity for the isolation of peptides with multiple phosphorylation sites [56] whereas ZrO_2_ has been reported to perform better for single site phosphorylated peptides [57].

Whilst working through similar mechanisms, MOAC is now more commonly used. Furthermore, the use of MOAC columns has been demonstrated to be more stable [58] and more selective [59] than IMAC. Both techniques enrich for Serine, Threonine and Tyrosine phosphorylation sites at the ratios that they are present in cells (70 : 30 : 2, respectively). When the aim is to specifically analyse the phospho-tyrosine phosphoproteome, immunoprecipitation of peptides using anti-pTyr antibodies has been demonstrated to provide greater coverage of this particular sub-phosphoproteome [60].

For many phosphoproteomic workflows, the sample preparation procedure is more laborious than the assay setting the biological context of the experiment. Phosphoenrichment is a particularly laborious task if done manually. However, the development of robotic liquid handlers for this purpose essentially solves this issue and also help with the reproducibility and throughput of the technique [61,62].

### Label-free Tandem mass spectrometry data acquisition

Peptide identification and quantification from MS data require the use of computationally demanding analytical pipelines. Traditionally, proteomic approaches involve acquiring MS2 spectra using data-dependent acquisition (DDA) methodologies which select the most abundant *m*/*z* peaks in MS1 spectra for fragmentation at a given time in the LC separation. The selection for MS2 is based on precursor ions’ abundance ranking in MS1 spectra. Investigators will set the number of peptides which will be selected by DDA based on experimental considerations and the scan rates of the instrument that is being used — modern mass spectrometers have scan rates that range from 10 to 100 Hertz. These selected ions are then fragmented and the resulting MS2 spectra are used to identify the analyte with a database search that aims to match fragment spectra to the theoretical fragmentation of all proteins in databases or to spectral libraries [63–67]. This approach allows sensitive real-time detection of peptides/phosphopeptides. The MS1 scan dependency enables the acquisition of spectra of sufficient quality and quantity for deep phosphoproteome coverage [30,32,68,69].

**Figure 2.**
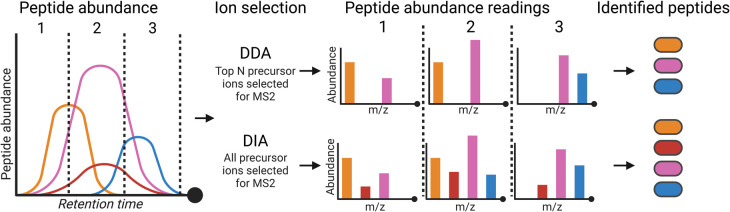
Overview of DIA and DDA approaches in proteomics. Peptide abundance varies throughout the LC gradient and, as peptides elute from the column at specific time points, these are sampled by MS (represented as windows 1, 2 and 3 in the cartoon) and subsequently by MS/MS analysis. In data dependent acquisition (DDA) only the top N peptides (depending on instrument scan rates) are selected by abundance. This means that some peptides (e.g. the red ion in the cartoon) may be omitted from all windows and are not identified in DDA. This is contrary to data-independent analysis (DIA), where all peptide ions are fragmented and thus present in MS2 scans.

Whilst a proven useful approach, a limitation of DDA includes a bias towards more abundant peptides due to preferential selection of intense ions for MS2 analysis, and variability between the peptides that are selected for fragmentation in runs which can arise from minute scan time variations and other stochastic processes (Figure 2). These issues with undersampling lead to missing peptide identifications, a problem that increases with sample complexity [70]. To solve this problem, peptides and phosphopeptides identified in at least one LC–MS/MS run can be searched in other runs at the MS1 level. This approach consists of assembling a database (or library) of peptides identified in a given experiment; the elution profiles (extracted ion chromatograms) are then obtained for each of these peptides across all LC–MS/MS runs that are compared in the experiment, thus significantly alleviating the problem of undersampling in DDA [48,71–73].

Another way of reducing the impact that undersampling in DDA has on proteomic and phosphoproteomics data reproducibility involves the use of data independent acquisition (DIA) analysis [74–76]. Advances in computational processing tools and processing power have overcome the challenges which hampered the widespread application of this technique. DIA has the potential to enhance MS data pipelines and broaden reliable analyte quantification, particularly when used in combination with cutting-edge mass spectrometers with increased duty cycles aided by ion mobility ion optics [77,78]. During DIA acquisition, ions are not fragmented in an intensity-selective manner, rather all precursor ions within a selected *m*/*z* range are fragmented thus removing bias towards more abundant ions and reducing the stochasticity of MS2 ion generation (Figure 2). This approach has been demonstrated to decrease missing data points in proteomics datasets, although this comes at the cost of reduced MS2 spectral interpretability [79,80] requiring more complex computational analysis to deconvolute MS2 spectra into peptide identities.

Initially, DIA was not widely adopted in phosphoproteomic studies due to the requirements of mass spectrometers with fast scan rates, which are now becoming available, and the computational strain that accompanied the disentanglement of more complex fractionation spectra due to the fact that there is no direct link between single MS1 peaks and MS2 spectra. Recent developments in software for DIA data analysis [81,82] are enabling widespread use of the technique in proteome research, but the use of DIA in phosphoproteomics is less well documented, although we expect the technique to become widespread for this purpose now that robust software has become available. However, since most phosphoproteomics experiments have been performed using DDA, we focus on studies that used this approach henceforth. We refer the reader to the work of Zhang et al. [83] for further discussion on DIA.

### Assigning peptides to *m*/*z* spectra

Following data acquisition, the complex ion spectra must be resolved into peptide/phosphopeptide identities in order to identify proteins/phosphoproteins and phosphorylation sites. In database search methods, protein sequences in an appropriate database (e.g. Uniprot restricted to the organism analysed in the experiment) undergo *in silico* digestion and fragmentation to produce a reference of artificial peptide spectra, to which the experimental spectra is matched by a searching engine that also scores and ranks the identifications.

A widespread method for this is scoring against theoretical spectra using ‘shared peak count'. Pioneering algorithms using such a strategy include SEQUEST [67] which is based on an XCorr score that serves as a measure of similarity between acquired and theoretical peptide spectra. A limitation of the method was the speed at which XCorr is calculated which became a bottleneck for MS pipelines. Subsequent efforts to increase processing speed have included dot product computation of XCorr [84]. Further efforts have been built on to the SEQUEST algorithm, such as TIDE, which takes advantage software engineering approaches to further boost speed [85].

Other algorithms for database search in proteomics include the open source X!Tanden and Protein Prospector. The latter was developed in 1995 at the University of California, San Francisco to exploit the additional specificity of identification that can be obtained by reducing the mass error windows used to match experimental with theoretical peptides [65]. It has since then undergone many iterations with additional features added to the software suite [86] and continues to be commonly used today [87]. A further more recent development is a database search method known as MSFragger which was designed with the aim of increasing processing speed. Similar to SEQUEST (resulting in the tool Crux) [88] the developers of MSFragger added a peptide database index to increase pipeline speed, however, in contrast to Crux which solely sorted peptides by mass, MSFragger additionally computes a theoretical fragment ion index, leading to improved efficiency compared with other tools [89].

In contrast to the algorithms described above, which use heuristic approaches, probabilistic algorithms, such as Mascot — widely considered the industry gold standard [66,90] — and Andromeda (search engine in MaxQuant) assign scores to peptides as the probability that the match between the experiment peptide spectra and theoretical spectra has occurred by chance. Mascot uses the MOWSE algorithm [91], whilst MaxQuant [92] offers flexibility allowing the researcher to tailor their analytical pipeline.

For phosphopeptides, there is the additional challenge of identifying the phosphosite location. These issues have been reviewed recently and therefore they will not be discussed here in depth [36]. Briefly, in cases where a peptide contains more than one serine, threonine or tyrosine residue in its sequence, confident phosphosite allocation requires ‘site-determining ions' to be generated from fragmentation spectra [93], which may not be present as a result of neutral loss during CID MS/MS. Different phosphorylated residues can also have different fragmentation behaviours depending on peptide residues which different scoring algorithms can be biased to [94]. To address this, scoring methods were developed, and these include the mascot delta score [94] and Ascore [93], though a wide range of tools exist [36,95]. More recently, MS/MS spectra prediction and DIA have been proposed as means to increase the accuracy of site localisation in phosphoproteomics experiments [96,97].

### Peptide verification

Proteomic identification results are adjusted for the probability of false discovery using a target-decoy method where results from searching artificial peptide sequences not found in a biological context (e.g. in a reversed protein database) are compared with those of the experimental (forward) search results. Characteristics of the ideal decoy sequence have been described by Gygi's group [98]. In brief, this relates to sharing characteristics with target sequences (residue distribution, similar length and numbers at both peptide and protein level, and importantly no shared peptide sequences). Many approaches exist for generating the decoy database, such as peptide reversal and random sequence generation. Caveats to these include the non-randomness of sequence reversal, and in contrast, random sequence generation will unlikely maintain relevant peptide characteristics such as the presence of repeated residues [99].

It has been demonstrated that simple target-decoy strategies lead to reduction in sensitivity due to asymmetry in protein matches relating to false artificial proteins and false target protein hits, resulting from the fact that incorrect target peptide matches could occur from both target proteins and decoy proteins, in contrast to decoy peptides [100,101]. To address this, Savitski et al. [102] developed the picked-target decoy strategy (picked TDS). This approach simply compares the scores of both the target peptide match and the decoy peptide match and picks the highest-scoring match for that target peptide. Using this simple strategy, the overestimation of false positives was reduced in the aforementioned study. Other efforts to address this issue include that of The et al. [101], who developed an approach known as the Picked Protein GroupFDR approach for protein group level FDR correction, resulting in 4% more protein identifications than the default approach in the MaxQuant pipeline [101].

A specific issue for phosphoproteomics is the estimation of false phosphorylation site localisation and the increase in search space that comes with including all the possible modifications on a given peptide in the search [95]. Efforts to address this include modified decoy search strategy, such as LuciPHOr, which uses each residue in the peptide as a phosphorylation site decoy, thus pitting candidate and non-candidate residues against one another [103]. Further work by Ramsbottom et al. [104] demonstrated that Alanine or Leucine residues make sensible decoy candidates.

## Bioinformatic tools and resources for phosphoproteomic data interpretation

### Phosphoproteomic analysis tools

A challenge in omics analysis is biological interpretation but the issue in phosphoproteomics is particularly intricate because of the impact that phosphorylation has on biology is often ambiguous — some sites increase the activity of the protein that is modified, but many are inhibitory while for most of them there is no functional information. To address this, two main computational approaches to derive biological insights from phosphoproteomics data are employed, which we term substrate-centric and kinase-centric. The substrate-centric approach involves considering the effects that changes in individual phosphorylation sites may have on the protein that receives the phosphorylation, whereas the kinase-centric method consists of deriving readouts of kinase activity by matching kinases to their substrates or downstream targets. Both methods may be combined to analyse a given dataset.

The substrate-centric approach needs information on the effect that a given phosphorylation site may have on the activity of the protein receiving these modifications. This is a challenge because, as discussed above, while some phosphorylation sites are well characterised, in most cases the biological function of phosphorylation is ambiguous. The low conservation of phosphorylation across species suggests that most sites are non-functional [105,106], but this view has been challenged [107]. Recently, machine learning was used to infer the phosphorylation sites likely to be functional [108], and progress is also being made in predicting the sign of regulation (i.e. whether the phosphorylation is activatory or inhibitory) [109].

As for the kinase-centric approach, kinase-substrate enrichment analysis (KSEA) and similar methods infer differences in kinase activities across samples by matching kinases to their substrates and quantifying their level of enrichment relative to background phosphorylation (by for example, z-score calculation and/or Kolmogorov–Smirnov testing) [17]. In the original study, KSEA-derived kinase activities showed high concordance to western blot data [17] and changed in cells treated with signalling perturbagens as expected. KSEA is based on the analysis of validated kinase-substrate relationships and thus provides a more reliable method of characterising kinase activity than investigating individual kinase phosphorylation sites [16–18,110] or approaches such as NetworKIN [111] which computationally predicts kinase substrates based on linear phosphorylation motifs.

Since the publication of KSEA other methods that use phosphoproteomics data to infer kinase activity have been published. These have been recently reviewed and will not be discussed here in detail [112]. All these methods require a knowledge of phosphorylation sites that are signatures of kinase activities. Therefore, the challenge that all these tools face is that the knowledge of kinase-phosphosite relationships is limited to the best-studied kinases, and for many of them there is a lack of knowledge of their downstream targets, thus precluding their analysis by this method. To solve this problem, Hijazi et al. used the known promiscuity of kinase inhibitors to map kinase downstream targets of less well-studied kinases. Kinase inhibitors developed to inhibit a given kinase also inhibit unintended enzymes, some of which are poorly studied. This was exploited to identify the combination of compounds that represent specific inhibitor fingerprints for given kinases. Phosphoproteomics of cells treated with the set of inhibitors in the fingerprint then revealed phosphorylation sites downstream of kinases [24]. The study added 6 000 new kinase-phosphosite relationships that may be used as the source of KSEA or similar methods to infer kinase activities from phosphoproteomics data. A version of KSEA that incorporates this and other databases as the source of kinase-phosphosite relationships can be accessed from github.com/CutillasLab/KSEA_plus [21].

Contrary to KSEA, which compares kinase activities across samples, other methods focus on ranking kinase activities within a sample. This information may be used for selecting the best kinase inhibitor to treat a given patient. Two of these methods have been developed: Kinase activity ranking using phosphoproteomics data (KARP) [113] and the inferred kinase activity (INKA) algorithm developed by the Jimenez laboratory [114]. INKA estimates kinase activity as a function of total kinase phosphorylation, phosphorylation of the kinase's activation loop and substrate phosphorylation, thus combining kinase and substrate-centric approaches. Using INKA, Cordeo et al. [25] selected kinase inhibitors for the treatment of acute myeloid leukaemia (AML) cell lines, highlighting its potential to inform treatment combinations.

### Empirical kinase network inference

Phosphoproteomic data have been used to reconstruct kinase networks. Methods for this purpose include the use of statistical frameworks combined with known substrate-kinase relationship data. An example is provided by work by the Saez-Rodriguez laboratory [115], who developed a method called Phonemes based on logic modelling of kinase inhibitor perturbation data through Gaussian mixture models for each phosphorylation site. This involved training models with a background network (built from known/predicted kinase-substrate relationships). Application of Phonemes identified CDK regulation by MTOR, which was verified experimentally. Using a complementary approach, Clarke et al. [116] developed a network-based tool to link expression signature to upstream kinase networks. Another strategy developed by Yilmaz et al. [117] uses a heterogeneous network model, which uses circuit-based propagation in an attempt to bridge missing network connections that can arise due to under sampling of the phosphoproteome, an inevitable issue due the large number of yet unknown kinase-phosphosite interactions [118] and under sampling of phosphorylation sites that can occur in MS experiments. Thus, this approach takes advantage of current network knowledge to fill in potential gaps in the kinome network under the assumption that biological events stemming from network action will originate from associated sites. However, this may introduce some bias into new explorations due to reliance on well-characterised signalling nodes. Other groups have focussed on using KSEA to provide a global analysis of kinase signalling. Ochoa et al. [119] compiled 41 studies including drug perturbation data for 399 kinases to track kinase–kinase relationships through monitoring the activity of a broad range of kinases in response to inhibitor treatment.

## Current challenges of kinase-phosphosite mapping

### Overcoming bias towards the known phosphoproteome

A major hurdle in phosphoproteomics is the low of coverage of known kinase-phosphosite interactions. Indeed, it is estimated that 87% of listed kinase substrates are phosphorylated by 20% of known kinases, a distribution that seems unlikely due to large number of shared substrates between kinases [118]. This bias is propagated by the fact that targeted functional assays are lengthy and time consuming, along with the problem of confirmation bias that can exist within scientific research which is present in phosphosite databases. To address this, Hijazi et al. [24] used a chemical phosphoproteomics approach to identify direct and indirect kinase-phosphosite relationships. More recently. Johnson et al. used positional scanning peptide array analysis (PSPA), consisting of *in vitro* assays with peptide pools and individual kinases and ATP, to determine motifs determinants of kinase-substrate specificity, primarily via the negative selection on the substrate's side. Furthermore, the authors found a third of the sites tested lacked such residues, and were therefore shared with a large number of kinases. This highlights substrate availability and other kinase regulatory mechanisms’ importance in coordinating specific signalling responses [120].

To document kinase-substrate relationships, there are many dedicated databases for recording such information. One of the largest databases is PhosphositePlus [121], which consists of curated information on protein PTMs for human, mouse and rat phosphoproteomes. Another database known as Signor, is also generated from curated literature of phosphorylation site information, but is stored in a directed graph format, the goal being to associate PTM to effect/function [122]. Another recent and useful metadatabase for the analysis of phosphoproteomics data is Omnipath [123]. Kinase-phosphosite relationships identified by chemical phosphoproteomics are provided in the ChemPhoPro database [24]. These are invaluable resources for the community, and the incorporation of curated experimental data from the literature, as these become available in the future, will result in more accurate signalling network construction approaches based on phosphoproteomics data.

Studies that used perturbation data can be a good source of new kinase-phosphosite relationships and also reveal kinase-kinase connexions. Perturbagens may be siRNA [124] or kinase inhibitors [24]. Bodenmiller et al. performed a kinome/phosphatase-wide genetic screen in yeast, where they individually perturbed kinase/phosphatases in yeast prior to MS analysis. They demonstrated that the number of indirect phosphosites far outnumber the predicted direct phosphosites and the interconnectivity of signalling architectures. On the other hand, as discussed above, the kinase inhibitor screen by Hijazi et al. relied on the promiscuity of kinase inhibitors to identify downstream targets of poorly studied kinases. The advantage of siRNA is its specificity, but it has the drawback that it requires long treatments times, and therefore changes in gene expression also contribute to the observed changes in phosphorylation. This does not occur in cells treated with kinase inhibitors for short times, but these approaches need complex algorithms to link kinase inhibitor specificity with their effects in the phosphoproteome. Despite these caveats, these large-scale screens, combined with mass spectrometry-based phosphoproteomics could contribute to a more balanced annotation of less well-explored kinases in commonly used reference databases.

### Data variability

One of the perceived drawbacks of phosphoproteomics data is that the high instability of phosphorylated peptides, due to their rapid degradation by phosphatases and high pH, contributes to noise and variability in such datasets. For phosphoproteomics data to be clinically meaningful, it has to be resilient to the highly dynamic nature of kinase signalling, which makes this technique susceptible to environmental changes. Responses to kinase inhibitors have been characterised minutes after treatment, in *in vitro* models [125] and in cancer cells left in ambient conditions, which resulted in the activation of stress and autophagy signalling pathways [126]. However, the activation of these pathways was reduced using simple measures (by placing cells on ice prior to the lysis procedure). In addition, phosphoproteomics experiments carried out in separate laboratories show differences in peptide coverage, in addition to intra-laboratory variation from different staff members. This highlights the reactivity of phosphorylation activity and the requirement for robust sample preparation protocols to create reliable datasets. However, these issues also greatly impact other omics methodologies such as single-cell RNA-Seq, RNA-Seq and CHIP-Seq [127–129].

The impact of batch effects has been greatly diminished in omics datasets due to improvements in their throughput, which allows characterising a wider range of biological conditions, improving signal-to-noise ratio and ultimately separating variation of biologically relevant changes from data noise [128,130]. Recent advances in automation have also allowed for the development of robust high throughput desalting and phosphoenrichment procedures, requiring minute protein amounts and allowing more samples to be processed in shorter times and improving reproducibility. These advancements in turn have made phosphoproteomics approaches more suited for use in translational and clinical investigations.

## Applications of phosphoproteomics in cancer research

**Figure 3.**
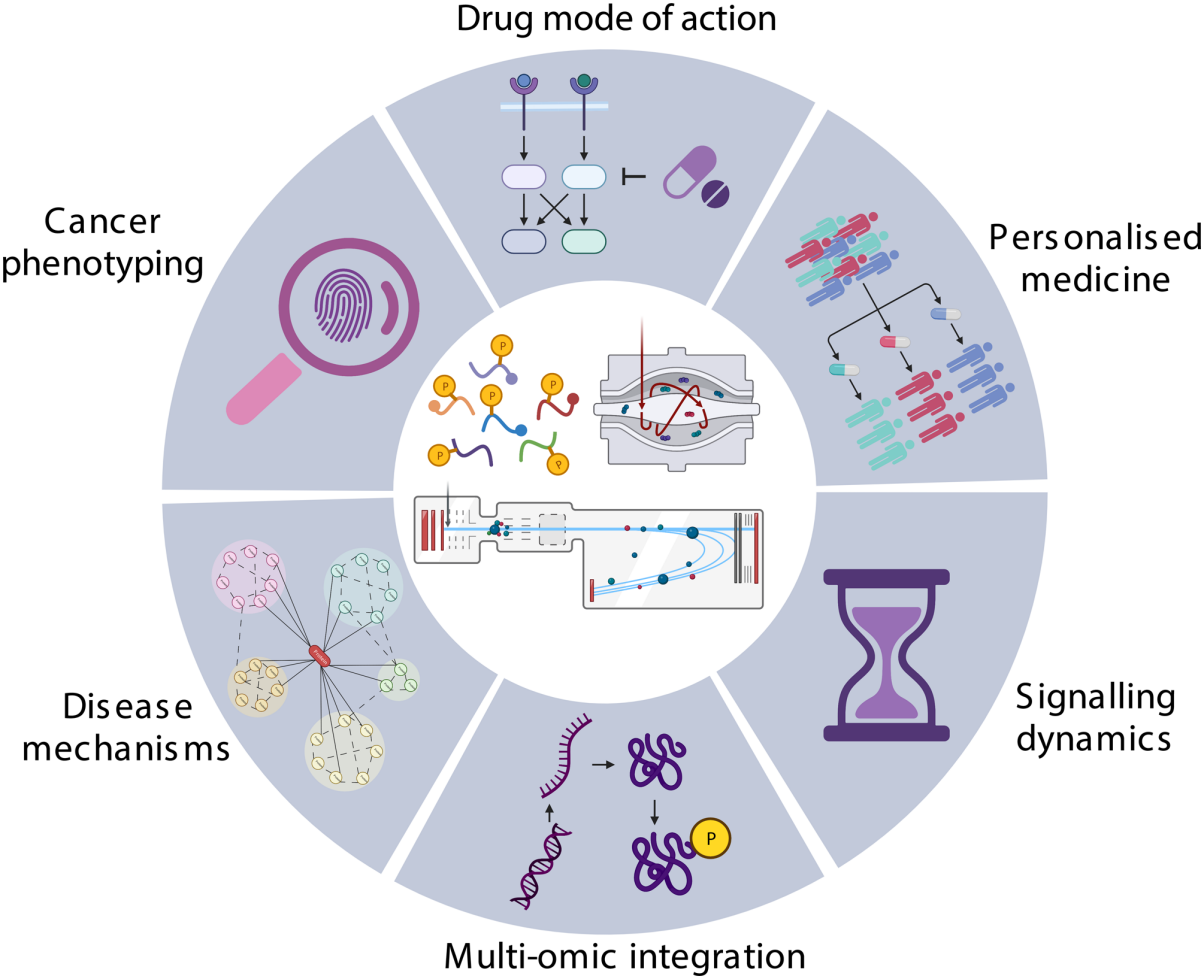
Application of phosphoproteomics in cancer research.

Phosphoproteomics has numerous uses in cancer research (examples are shown in Figure 3). Among other applications, it has been used to rationalise cancer phenotypes, as a readout of gene knock-out experiments, to investigate the mode of action of anti-cancer drugs, and as a source of biomarkers for precision medicine. Below we give examples of studies that illustrate the use of phosphoproteomics in these research areas.

### Rationalisation of cancer phenotypes using phosphoproteomic data

The first application of phosphoproteomics followed by KSEA focused on the investigation of determinants of drug response phenotypes in AML cell lines and primary samples treated with PI3K inhibitors [17]. Contrary to expectations, cells with higher PI3K/MTOR pathway activity were not more sensitive to PI3K/MTOR dual inhibitors than cells with lower activity of this pathway. Instead, activities of kinase in parallel pathways — namely PKC and ERK — were increased in resistant cells, showing that measuring the activity of compensatory pathways is needed to fully rationalise why some cancer cells respond to kinase inhibitors whereas others do not. This phenomenon was also observed in cells treated with trametinib, a MEK inhibitor. Although cells with high MEK/MAPK pathway activity responded better to trametinib than cells with low pathway activity, other compensatory pathways (including FLT3 mutations and STAT5 activation) were increased in cells that, despite activating MEK/MAPK, were resistant to inhibitors this pathway [14]. More recently, in a pan-cancer study, KSEA of phosphoproteomics data was used to estimate activities of 218 kinases across 1 110 tumours and 77 cell lines [131]. These data were matched to the activities of 292 transcription factors as well as genetic mutations and responses to therapy. An interesting observation was that overall kinase phosphorylation is, for some kinases, significantly associated with their extent of activation, as noted in a previous study [24], while TF activation is associated with their expression at the protein level. Of note, it was found that loss of function mutations was not on the whole associated with the activation of downstream signalling cascades, suggesting that, in most cases, feedback loops that suppress excessive pathway activation [132] are maintained in cancer cells. However, the activities of frequently dysregulated proteins were found to be associated with drug responses [131], thus suggesting that these could be used to determine prognosis. Taken together, these studies illustrate the complex nature of kinase signalling regulation, which underpin the idea that proxies of pathway activities (such as genetic mutation or protein expression) often fail to accurately reveal the extent of oncogenic pathways activation in cancer cells.

Several other studies have applied phosphoproteomics methods to investigate the mode of action of specific anti-cancer drugs. A study by Kazi et al. [133] applied phosphoproteomics analysis to KRAS mutant dependent and non-dependent pancreatic cancer cell lines to reveal a key role of CDK kinases in KRAS dependency. Following these results the authors validated their finding through mining CRISPR–cas9 dependency data, drug screens and western blot endpoints showed that targeting CDK hyperactivation may be an effective way of treating KRAS mutant pancreatic cancer. Similarly, Lee et al. [134] applied this approach to gauge the signalling shifts involved in the development of resistance against an EGFR/HER2 inhibitor (lapatinib) in human gastric cancer cells (SNU216 cells). Comparison between lapatinib resistant and parental SNU216 cells identified a MET-derived signalling cascade that increases the activity of the PI3K/AKT and MAPK/ERK signalling axes. Another example of phosphoproteomic interrogation is provided by Vye et al. [135], who used SILAC mass spectrometry to identify two distinct resistance mechanisms to both pazopanib and dasatinib in A204 cells, revealing that dasatinib-resistant cells upregulated insulin receptor/IGF-1R signalling as a compensatory pathway. An elegant study by Emdal et al. [19] extended this to primary AML samples where phosphoproteomic analysis of responders and non-responders to selinexor highlighted AKT as a combination target for resistant cells.

### Phosphoproteomics to elucidate disease mechanisms

Phosphoproteomics is also a powerful tool to provide mechanistic insights into cancer cell biology and signalling in different tumour types. Examples of discoveries made by KSEA include the identification of protein kinase A as a mediator of an increasing cell metabolism in tumours with low FAK expression in cancer-associated fibroblasts [7], the identification of the MAPK-ROCK2 pathway being activated in cancer cells by loss of β-integrin in mural endothelial cells [136] and the discovery of MAPKAPK2 as a kinase downstream of MTOR that regulates the senescence secretome [137]. In a recent study, phosphoproteomics and KSEA showed that targeting the lysine demethylase LSD1 in AML cells activates the MEK/MAPK pathway, while inhibiting PI3K/AKT signalling. Consequently, LSD1 inhibition produced a signalling switch that primed cells for MEK treatment [138].

In a different study, Tamir et al. applied functional kinome screens for target stratification followed by phosphoproteomic interrogation. The authors performed a gain-of-function arrayed screen whereby 385 kinases and kinase-associated proteins where overexpressed in HEK-293T cells. Using this approach, an unexplored kinase BRSK1/2 showed a pivotal role in NFR2 transcription factor regulation, relevant in cancer and neurodegenerative disease. Subsequent analysis using TMT labelled phosphoproteomics of transfected BRSK1 and BRSK2 cells revealed an increased number of key phosphosites in BRSK2 transfected cells, suggesting a more central signalling role than its BRSK1 homologue [139].

### Precision medicine

The phosphoproteome stands to provide a rich source of biomarkers of drug responses that can complement the information provided by genetics to advance the field of precision medicine. As an example of study, Liu et al. [140] were able to define molecular-based subtype profiles for cancer patients and signatures which were used to propose candidate drugs for more informed treatment. The volume and complex nature of biomarkers identified will require machine learning approaches to make sense of this wealth of data for advancing precision medicine and this is a highly active area of research. For example, Gerdes et al. [22] developed a machine-learning workflow which used phosphoproteomic data to rank the most effective treatments based on phosphoproteomic profiles. To this end, the authors used a novel feature selection strategy and an internal distance metric to narrow down selected markers, thereby accurately predicting drug responses in cell lines obtained from different lineages. Importantly, the models created by Gerdes et al. [141], also predicted drug responses in an independent study focusing on cholangiocarcinoma cell lines and primary samples. These proof-of-principle studies support the integration of machine learning-based precision medicine [142], although more work is needed for the confident roll out of these methods in the clinic. Despite the remaining challenges, it is clear that the phosphoproteome will be an invaluable source of information to guide the next generation of targeted and personalised therapies.

### Unpacking kinome dynamics

**Figure 4.**
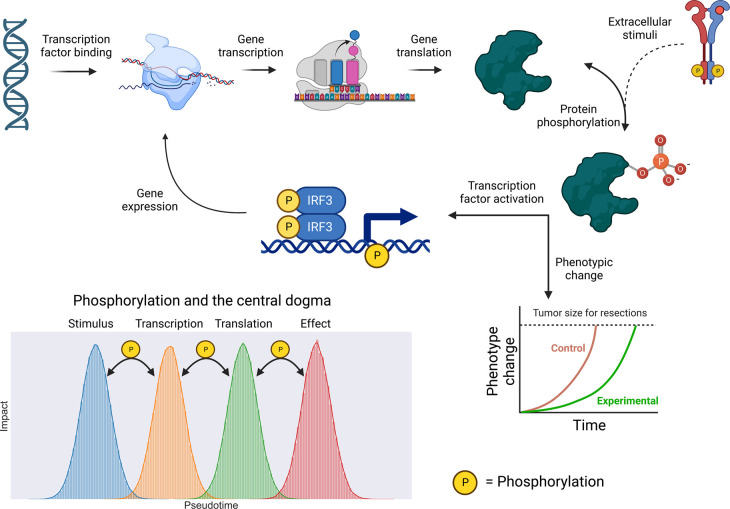
The Phosphoproteome acts as the cell's sensor, regulator and effector. Protein phosphorylation has diverse impacts and roles in cell biology. Effects relayed by increase expression of exomic genes often yield protein products whose activity is regulated by phosphorylation. A prime example is transcription factors (here uses IFN regulatory factor 3 (IRF3) as an illustrative example), whereby phosphorylation can lead to increased expression of genes and drive as well as regulate changes in cell phenotype, which can occur due to external or internal stimuli. Changes in the phosphoproteome can originate from extracellular/intracellular cues and impact all stages of the central dogma in biology (lower left panel).

Signalling is a dynamic process and perturbation with agonists or antagonists induces waves of kinase activation that are specific for given pathways and cell types. Thus, sampling time should be carefully considered as targeting distinct kinase signalling nodes can have temporally distinct influences on cell phenotypes [143]. Analysis techniques tailored for temporal analysis are being developed in attempt to increase time-series understanding of kinases [144]. As depicted in Figure 4, the importance of phosphorylation can be felt across the gene expression process illustrating complex and non-linear timelines in terms of relaying signals from/to the transcriptome/genome. For example, an in-depth view of the Drosophila phosphoproteome in combination with proteomics/transcriptomics, identified key kinases involved in the organism's circadian rhythm [145]. Understanding key checkpoint kinases could be used for targeting disease phenotypes. Alternatively, these temporal phosphoproteomics could pin point key signalling effectors in the acquisition of disease phenotypes and enable increasing understanding of how successful treatment leads to transition from a disease to disease-free state. As an example, an insightful study [146] used phosphoproteomics to compare kinase activation in HER2-postive gastric cancers before and after treatment with the HER2 antibody inhibitor trastuzumab. By comparing phosphoproteomic analysis of gastric cancer biopsies before and after treatment, the authors were able to gain insight into the impact of the HER2 antibody on the phosphoproteome enabling the discovery of new response biomarkers.

### Phosphoproteomics’ role in the omics landscape

Phosphoproteomics data have had an important role to play in the multi-omic profiling of primary clinical samples. A successful initiative known as the clinical proteomic tumour analysis consortium (CPTAC) was established in 2011 and since become a trove of proteomic and genomic data from primary tumour samples. These and other studies have provided invaluable datasets from different cancer types including breast [147], hepatocellular [148], ovarian [149], haematological [14,150], lung [151] and colorectal [152] among others. These studies are providing a wealth of information of disease subtypes and opportunities to identify new kinase drug targets [149]. As an example, Casado et al. [153] found a phosphoproteomic signature that defines two biologically distinct forms of KMT2A rearranged AML (also known as MLL-AML). This is significant because MLL-AML was considered to be a homogenous disease from a biological standpoint. Contrary to this view, phosphoproteomics data revealed two MLL-AML subgroups that differ in the phosphorylation of several proteins involved in KMT2A-mediated regulation of gene expression, including DOT1L. These differences in the biochemistry of MLL-AML cells translated to differences in responses to genotoxic and other drugs, suggesting that the identified signature may be used to select patients for therapy.

Integration of multi-omic data is key for making the most of such complex datasets but, until recently, software for this purpose was not available and researchers have used simple approaches such as the correlation between protein and RNA expression [154]. In response to this, computational tools have been developed to integrate different omics datasets. One such tool includes MiNETi [155], a computational pipeline that integrates proteomics, phosphoproteomics and transcriptomics to construct a multi-network spanning graph. This is achieved by sequential network updates starting with a proteomics-based PPI network, updated with phosphorylation network data and finally transcriptional networks [155]. Other multi-omic analysis tools include COSMOS, which also connects transcription factors and kinase activity. COSMOS performs this by constructing prior knowledge network spanning the multi-omic space [156].

## Conclusions

There is a clear role for phosphoproteomics to play in the interrogation of oncogenic mechanisms and for drug development. Improvements in technology offer new opportunities to derive insights into cellular signalling and regulatory biology with unprecedented depth, accentuating the interwoven nature of signalling mechanisms. However, challenges remain in the field of phosphoproteomics. For example, research resources for phosphoproteomics lag behind those of nucleotide-based ‘omics' due to the highly specialised nature of the technology and the need for expensive equipment, and the potential to translate phosphoproteomics biomarkers into clinical assays has not yet been demonstrated. In the future, further advances will come from improvements in mass spectrometers with enhanced sensitivity and shorter duty cycles that allow the application of new forms of data analysis (e.g. DIA) and from the development of computational methods to better interrogate phosphoproteomics data in the context of kinase-phosphosite relationships and the biological function of the phosphorylation sites that can be profiled with this technique. Together, these developments are enabling the analysis of the phosphoproteome as a means to tailor drug treatments to patients, for drug development and for the elucidation of mechanisms that govern normal and disease biology.

## Abbreviations

AML: acute myeloid leukaemia
CID: collision-induced dissociation
DDA: data-dependent acquisition
DIA: data-independent acquisition
ETD: electron transfer dissociation
HCD: higher-energy collision dissociation
IMAC: immobilised metal affinity chromatography
INKA: inferred kinase activity
KSEA: kinase-substrate enrichment analysis
MOAC: metal oxide affinity chromatography
PPI: protein–protein interaction
PTMs: post-translational protein modifications
SILAC: stable isotope labelling by/with amino acids in cell culture
TiO_2_: titanium dioxide
TMT: tandem mass tag
TOF: time of flight
